# Case of lead poisoning secondary to intake of herbal medicine for diabetes mellitus in a tertiary care hospital in Kerala

**DOI:** 10.1530/EDM-23-0066

**Published:** 2024-06-10

**Authors:** Junith Thomas, Rohini Sebastian, C R Anil Kumar, Aboobacker Mohamed Rafi

**Affiliations:** 1 Department of Transfusion Medicine, Jubilee Mission Medical College, Thrissur, Kerala, India; 2 Department of Pathology, Jubilee Mission Medical College, Thrissur, Kerala, India; 3 Department of General Medicine, Jubilee Mission Medical College, Thrissur, Kerala, India

**Keywords:** Geriatric, Female, Asian - Indian, India, Pancreas, Thyroid, Diabetes, Unusual effects of medical treatment, June, 2024

## Abstract

**Summary:**

Although most published cases of lead poisoning come from occupational exposures, some traditional remedies may also contain toxic amounts of lead. Here, we report the case of a 58-year-old female who presented with abdominal pain, generalized tiredness, and decreased food intake, with anemia and elevated levels of lead. The patient was found to be taking herbal capsules for diabetes prior to the presentation. This case highlights the need for increased awareness that some herbal remedies may contain potentially harmful levels of heavy metals, and people who use them are at risk of developing associated toxicities.

**Learning points:**

- Individuals who support traditional medicine often incorrectly believe that herbal remedies for diabetes are free from side effects, leading them to favor these treatments over contemporary medications.
- Herbal medications are freely available online, even without a prescription.
- The accessibility of herbal medicines without prescriptions, coupled with the false belief in their lack of side effects, misleads educated individuals toward quackery treatments. Misinformation spread via social media exacerbates this issue.
- Heavy metals are present in toxic levels in the drugs, causing complications.
- Lead is the most common heavy metal found in such herbal medicines.
- Lead poisoning leads to anemia and other systemic complications which could have been fatal if not found in time.

## Background

Ayurvedic medicine is a traditional system native to India. This system stresses the use of natural plant-based medicines. Minerals including sulfur, arsenic, lead, copper, and gold are often added to formulations with the belief that these metals are essential components of vital molecules within the human body. Some ayurvedic preparations have been found to contain lead and/or mercury at 100 to 10 000 times greater than acceptable limits. Thus, without sufficient public awareness, the risk of heavy metal exposure in individuals taking these supplements is quite high. Here, we present a case of lead poisoning secondary to ingestion of ayurvedic medicine  – Sheopal’s Herbal Diabetes Care Capsule procured through the internet.

## Case presentation

A 58-year-old female with a history of type II diabetes and hypothyroidism presented to the out-patient department with complaints of generalized tiredness and decreased food intake for 2 weeks. She also complained of abdominal pain for 2 weeks after eating. It was not associated with nausea or vomiting. She has no fever, loose stools, breathing difficulty, melena, or hematemesis. She was comparatively healthy prior to this illness. She was previously on metformin, which she had stopped 2 months back. She is on thyroxine tablets for hypothyroidism, which was under control.

On examination, she was conscious and well-oriented. Pallor was present with no icterus, cyanosis, clubbing, lymphadenopathy, or edema. Vitals recorded were within normal limits. No abnormality was detected in the systemic examination. The oral examination a revealed desquamation of the tongue and oral mucosa. She was admitted for further evaluation.

## Investigation

The laboratory tests were done and are as follows (Table 1): the hemogram revealed a hemoglobin of 7.5 gm/dL, a hematocrit of 23.1% with a reticulocyte count. White blood cell, differential count, platelet count, renal function, and liver function tests were normal. HbA1c on admission was 9%, suggestive of poor glycemic control. Thyroid function tests were within normal limits.

**Table 1:** Laboratory tests.

Test	Value	Reference range
Hb, g/dL	7.5	12–14
HCT, %	23.1	36–47
MCH, pg	25.7	25–35
MCHC, %	32.3	32–36
Platelet count, ×10^3^/μL	300	150–400
Total count, ×10^3/^μL	6.31	4–11
RDW, %	15.3	11–13.5
Reticulocyte count, %	7.94	0.2–2.0
Blood urea, mg/dL	11	10–45
Creatinine. mg/dL	0.7	0.66–1.3
Sodium, mEq/L	136	136–145
Potassium, mEq/L	3.8	3.5–5.0
Serum protein, g/dL	7.4	6.0–8.0
Albumin, mg/dL	4.2	3.5–5.2
A/g ratio	1.3	0.9–2.0
Globulin, g/dL	3.1	2.4–4.0
SGOT, U/L	139	0–35
SGPT, U/L	146	0–40
Alkaline phosphatase, U/L	156	30–120
Bilirubin total, mg/dL	1.4	0.3–1.2
Bilirubin direct, mg/dL	0.1	0.0–0.2
Iron, μg/dL	182	37–171
Ferritin, ng/mL	264	11–306
TIBC, μg/dL	165	265–497
RBS, mg/dL	**137**	80–160
PPBS, mg/dL	**174**	100–165
HbA1c, %	**9**	4–6.5
TSH, μIU/mL	**4.45**	0.38–5.33
T3, ng/mL	**0.92**	0.87–1.78
T4, μg/mL	**15.3**	4.8–15.6

## Treatment

**Figure 1:**
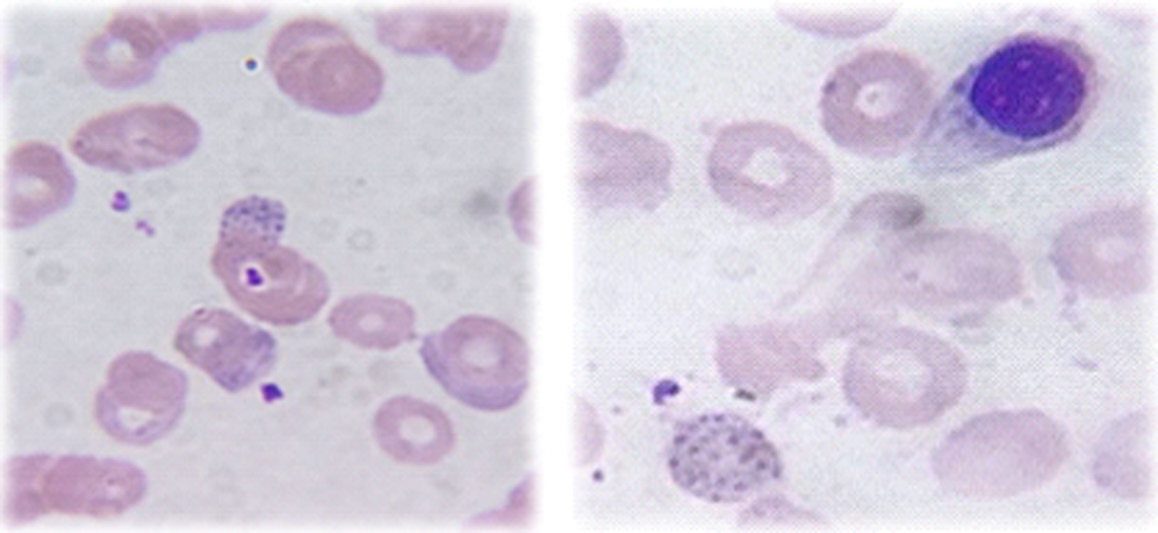
Peripheral blood smear.

**Figure 2:**
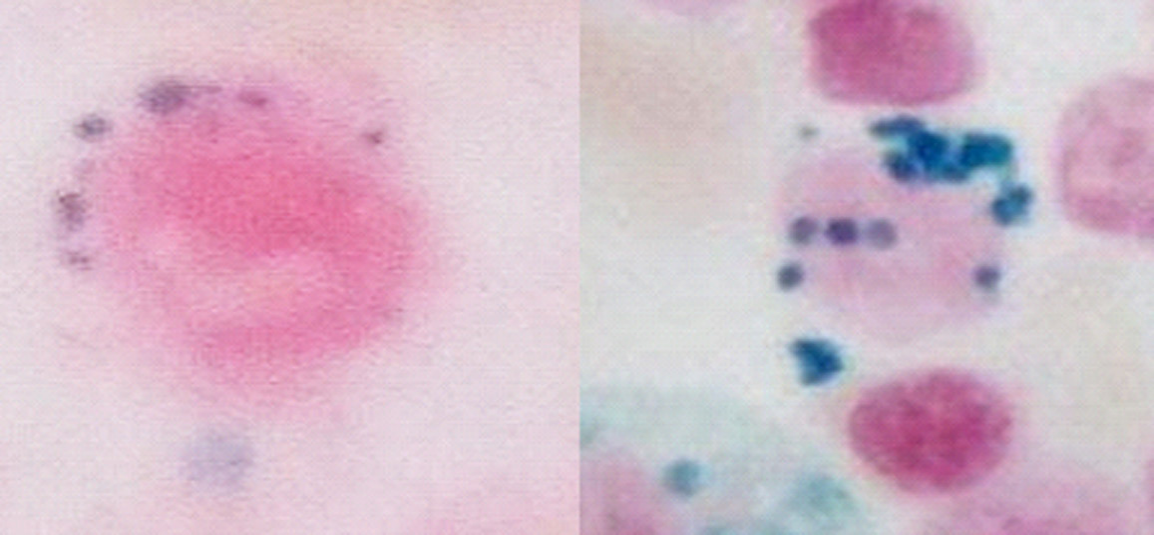
Ring sideroblast.

A final diagnosis of lead poisoning due to herbal supplementation was made. The patient was instructed not to take that herbal medication further. She was referred for chelation therapy. She was started on BAL, and later shifted to d-penicillamine. She became symptomatically better. Her Hb level improved and was 10.1 gm/dL at discharge. The USG abdomen showed no abnormalities. Careful examination of the peripheral blood smear revealed the presence of coarse basophilic stippling, normoblasts, and polychromasia, along with normocytic normochromic anemia (Fig. 1). Bone marrow examination showed erythroid hyperplasia with many ring sideroblasts in the aspirate, demonstrated by Perl’s Prussian blue stain (Fig. 2). This led to further questioning of the patient for any heavy metal exposure. She revealed that she had been taking an herbal medicine procured through the internet for glycemic control. She had been taking the medicine for a period of 1.5 months and had stopped prior to admission. Considering the findings of Basophilic stippling in the blood smear, ring sideroblasts in the bone marrow, elevated serum iron, low TIBC, and a history of intake of herbal medications, patient samples were sent for lead and zinc estimation. Significantly high levels of lead were found in the blood – 121.20 µg (reference range: <25 µg) and urine – 400.2 µg/dL (reference range: <80.0 µg/dL) of the patient (Table 2). Considering the patient’s age and to rule out other hematological conditions, a bone marrow evaluation was also done which suggested a possibility of acquired sideroblastic anemia attributed to drug-induced lead or zinc poisoning and a possibility of myelodysplastic syndrome with ring sideroblasts.

**Table 2:** Lead and zinc levels.

Test name	Result	Reference range
Blood lead, μg/dL	121.20	<25.00
Random urine lead, μg/dL	400.2	<80.0
Blood zinc, μg/dL	89.84	65.0–256.0
Lead (herbal capsule), ppm	40657.71	10*

*Permissible limit.

The drug sample in the form of capsules was sent for lead estimation to the state drug analyst revealing a very high amount of lead 40 657 ppm (permissible limit as per API: 10 ppm).

## Outcome and follow-up

She was kept on d-penicillamine orally for 6 weeks. Her blood and urine lead levels were retested and had come down to a normal level, with a peripheral blood smear showing mild normocytic normochromic anemia only.

## Discussion

Exposure to lead through ingestion or inhalation can occur from contaminated air, water, soil, food, and consumer products (1). Lead is stored in the blood, bone, and soft tissues, including the brain, spleen, kidneys, liver, and lungs (1). Like many other heavy metals, the presence of excess levels of lead leads to the production of free radicals, which subsequently causes oxidative damage to cellular components, including DNA and cell membranes (2). As an electropositive metal, lead has a high affinity for negatively charged sulfhydryl groups, resulting in the denaturation of enzymes such as delta-aminolevulinic acid dehydratase (ALA-D) and ferrochelatase, both of which are important for heme synthesis. The disruption of heme synthesis leads to the accumulation of free erythrocyte protoporphyrins. Inhibition of pyrimidine 5′-nucleotidase can prevent the degradation of ribosomal RNA in red blood cells leading to basophilic stippling on a peripheral smear, a classic finding that can be apparent at blood lead levels (BLLs) of ~50 μg/dL (3).

Symptoms of adult lead poisoning are variable and include abdominal pain, nausea, constipation, anorexia, fatigue, decreased libido, headache, irritability, arthralgias, myalgias, and anxiety. The current reference range for acceptable BLLs in healthy individuals without excessive exposure to environmental sources of lead is <10 μg/dL for children and <25 μg/dL for adults. Management varied widely and included oral chelation with d-penicillamine or meso-2,3-dimercaptosuccinic acid, or intravenous infusions of Ca EDTA, Na-EDTA, or dimercaprol. In some instances, combination therapy was administered (4). Chelation therapy should be initiated when the BLL is >80 μg/dL in asymptomatic individuals and >50 μg/dL in symptomatic adults and should be continued until the BLL is <50 μg/dL (5).

In the case presented here, timely diagnosis through a detailed evaluation of the peripheral smear, along with questioning and identification of the source of exposure, were critical in preventing the long-term consequences of lead poisoning. Enhancing public awareness about the harmful effects of seemingly innocuous herbal supplements is essential for the prevention of heavy metal poisoning.

## Declaration of interest

The authors declare that there is no conflict of interest that could be perceived as prejudicing the impartiality of the study reported.

## Funding

This work did not receive any specific grant from any funding agency in the public, commercial, or not-for-profit sector.

## Patient consent

Written informed consent for publication of the clinical details and clinical images was obtained from the patient.

## Patient’s perspective

The patient felt that we have picked up a real problem and the public and other clinicians must be made aware of the possibility of heavy metal poisoning, especially lead poisoning, while taking herbal medications. The patient is also keen that the government or other regulatory agencies should check the quality of drugs that are made available on the market.

## Author contribution statement

JT wrote up the case report; RS examined the peripheral smear; CRAK was involved in oversight of this case; AMR evaluated the patient and supervised the overall well-being of the patient and followed up the case report write-up.
